# Echocardiographic evidence of left ventricular untwisting-filling interplay

**DOI:** 10.1186/s12947-020-00190-6

**Published:** 2020-02-19

**Authors:** Amir Hodzic, Damien Garcia, Eric Saloux, Paula A. B. Ribeiro, Amélie Ethier, James D. Thomas, Paul Milliez, Hervé Normand, Francois Tournoux

**Affiliations:** 1 Department of Clinical Physiology, INSERM COMETE, Normandie Univ, UNICAEN, CHU de Caen Normandie, 14000 Caen, France; 2 Department of Cardiology, Normandie Univ, UNICAEN, CHU de Caen Normandie, 14000 Caen, France; 3 Research Center of the Hospital of the University of Montreal (Centre de Recherche du Centre Hospitalier de l’Université de Montréal), Montreal, Canada; 4 CREATIS, CNRS UMR 5220, INSERM U1206, Université Lyon 1, INSA Lyon, Villeurbanne, France; 5 Department of Cardiology, Bluhm Cardiovascular Institute, Northwestern University, Chicago, USA

**Keywords:** Diastolic function, Untwisting rate, Color Doppler M-mode, Intraventricular pressure gradient

## Abstract

**Background:**

Left ventricular untwisting generates an early diastolic intraventricular pressure gradient (DIVPG) than can be quantified by echocardiography. We sought to confirm the quantitative relationship between peak untwisting rate and peak DIVPG in a large adult population.

**Methods:**

From our echocardiographic database, we retrieved all the echocardiograms with a normal left ventricular ejection fraction, for whom color Doppler M-Mode interrogation of mitral inflow was available, and left ventricular untwisting rate was measurable using speckle tracking. Standard indices of left ventricular early diastolic function were assessed by Doppler (peaks E, e’ and Vp) and speckle tracking (peak strain rate Esr). Load dependency of DIVPG and untwisting rate was evaluated using a passive leg raising maneuver.

**Results:**

We included 154 subjects, aged between 18 to 77 years old, 63% were male. Test-retest reliability for color Doppler-derived DIVPG measurements was good, the intraclass correlation coefficients were 0.97 [0.91–0.99] and 0.97 [0.67–0.99] for intra- and inter-observer reproducibility, respectively. Peak DIVPG was positively correlated with peak untwisting rate (r = 0.73, *P* <  0.001). On multivariate analysis, peak DIVPG was the only diastolic parameter that was independently associated with untwisting rate. Age and gender were the clinical predictive factors for peak untwisting rate, whereas only age was independently associated with peak DIVPG. Untwisting rate and DIVPG were both load-dependent, without affecting their relationship.

**Conclusions:**

Color Doppler-derived peak DIVPG was quantitatively and independently associated with peak untwisting rate. It thus provides a reliable flow-based index of early left ventricular diastolic function.

## Introduction

Left ventricular (LV) untwisting is a key component of diastolic function [1]. This untwisting motion of the LV on its longitudinal axis occurs with elastic recoil of the base and apex from their previous systolic positions. The untwisting generates a diastolic intraventricular pressure gradient (DIVPG), contributing to the suction of blood from the left atrium into the LV at low atrial pressure [2]. Over the last decade, the assessment of LV untwisting motion and DIVPGs has been of increasing interest in cardiac imaging since they were independently associated with LV relaxation and restoring forces [3–5]. Tissue Doppler and later speckle tracking were used to measure LV untwisting. Although it was initially considered promising when its performance and accuracy were compared to those obtained with reference methods like cardiac magnetic resonance imaging and sonomicrometry [6, 7], its use is not widespread in clinical practice because of technical challenges and poor reproducibility [8, 9]. Color Doppler M-mode echocardiography is a validated technique for non-invasive assessment of DIVPGs [10, 11]. This approach estimating the instantaneous local pressure gradient from the measure of the spatiotemporal distribution of blood velocities along the LV long axis could provide an overview of the LV diastolic function. Prior studies in large animals and small human cohorts have suggested a close physiological relationship between those two parameters of early diastole [12, 13]. However, Doppler-derived DIVPG has not been widely applied in the LV diastolic functional assessment. We were able to develop fully automated software to measure non-invasively DIVPGs. The objective of this study was to investigate in a large population of subjects without heart disease the quantitative relationship between peak DIVPG assessed by color Doppler M-mode and peak LV untwisting rate estimated by speckle tracking.

## Methods

The echocardiographic laboratory of the CHUM (Centre Hospitalier de l’Université de Montréal) has a research database in which healthy volunteers and patients have consented to participate, authorizing access to their clinical information. We retrospectively retrieved from this database all echocardiograms performed over one year and meeting the following criteria: 1) an LV ejection fraction (LVEF) ≥ 50%, 2) an available color M-Mode tracing in apical four-chamber view as usually acquired for the assessment of the propagation flow velocity, and 3) the appropriate short-axis views to measure the untwisting rate using speckle tracking. Studies with the following criteria were excluded: known history of cardiac disease, regional wall motion abnormality, moderate or severe valvular heart disease, atrial fibrillation, or paced rhythm. Cardiovascular risk factors, physical activity behavior, and medical history were abstracted from either the questionnaire linked to our research database or from their medical records. The transthoracic echocardiograms were performed using one of our commercially available ultrasound machines: Vivid E9, Vivid 7 or Vivid q (GE Medical System, Milwaukee, WI, USA), equipped with a 2.5 MHz transducer. Acquisitions were performed in left lateral decubitus during an apnoea on three consecutive cardiac cycles with a stable ECG signal. All measurements were archived offline on a remote workstation (EchoPAC version 112, GE, Horten, Norway).

A passive leg raising maneuver had been performed by lifting the subject’s legs to a 45° angle to assess the load dependency of DIVPG and untwisting rate measurements, evaluated during the maneuver after heart rate stabilization. Lifting the legs passively from the horizontal position induces a gravitational transfer of blood from the lower limbs toward the intrathoracic compartment resulting in increased cardiac preload.

### Routine echocardiographic analysis

LV function was evaluated according to the current guidelines of the American Society of Echocardiography [9, 14]. LVEF was calculated using the Biplane Modified Simpson’s method. LV dimensions and mass were measured from a parasternal long-axis view and normalized to body surface area. Left atrial (LA) volume was measured by the Biplane method of disks during end-systole and normalized to body surface area. LV diastolic function was analyzed by measuring peak early (E) and late (A) transmitral Doppler velocities, peak mitral annulus early velocity (e’) by tissue Doppler imaging (average of septal and lateral peaks), and color M-mode Doppler flow propagation velocity (Vp) with the slope of the Nyquist velocity isoline after adjusting the color Doppler baseline. The E/A, E/e’ and E/Vp ratios were calculated. Doppler parameters were obtained as the average value of three consecutive cardiac cycles.

### Speckle tracking imaging and left ventricular untwisting analysis

**Fig. 1:**
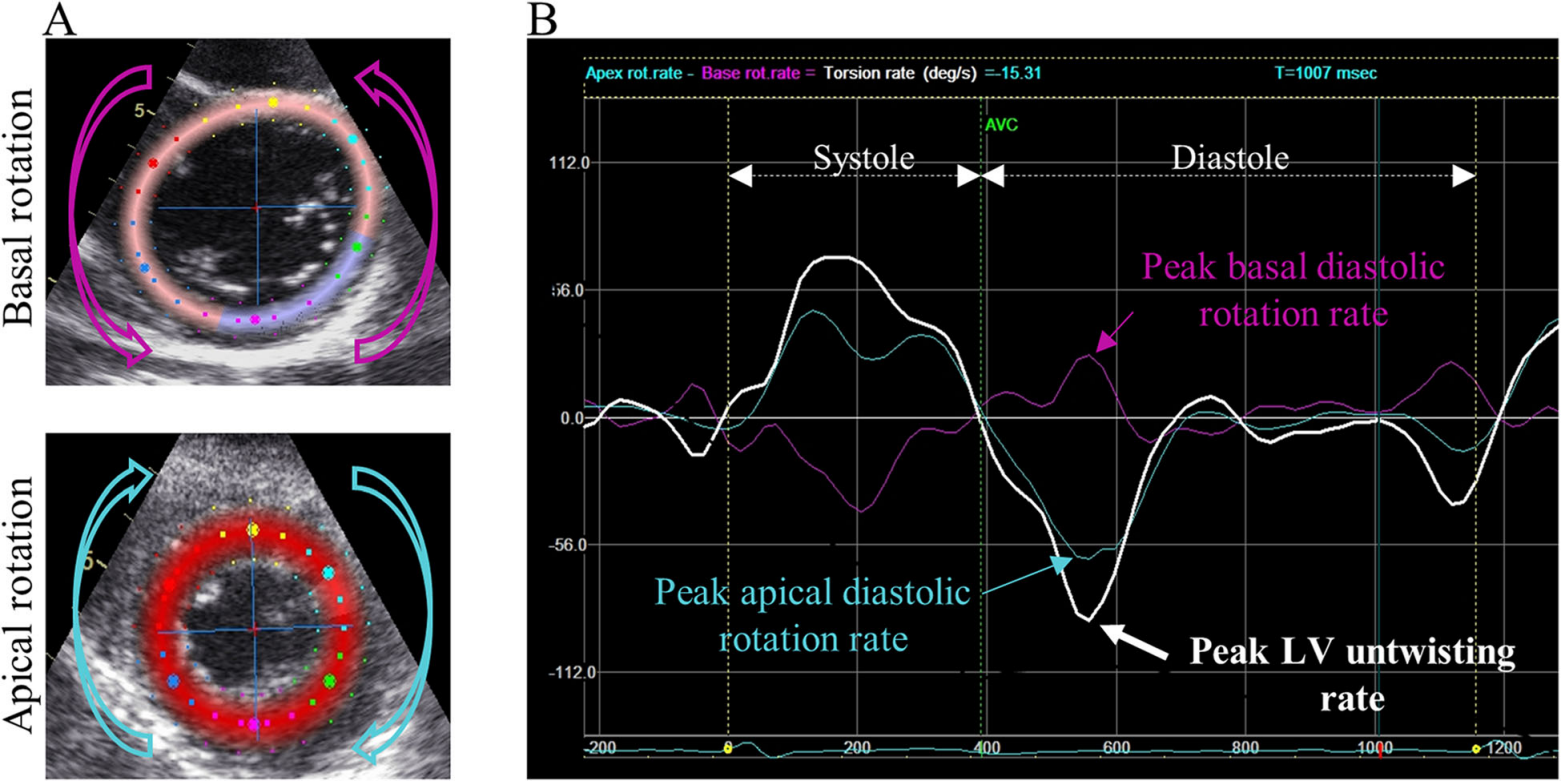
Measurement of left ventricular untwisting rate using speckle tracking **a**: Speckle tracking of left ventricular rotation in the short-axis view, at the basal and the apical levels (the arrows indicate the untwisting direction). **b**: Rotation rate curves from speckle tracking analysis.

LV untwisting rate was measured by two-dimensional speckle tracking on the parasternal short-axis views at the basal (mitral valve leaflets visible) and the apical (circular LV cavity, with no papillary muscle visible) levels, as previously described [7]. To ensure acceptable image quality, the frame rate was between 50 to 80 Hz. LV rotation was measured by displaying the rotation angle against time during the cardiac cycle in the apical and basal short-axis views. The averaged LV rotation and rotational velocity were obtained from six segments of each short-axis view, on three consecutive cardiac cycles. Peak untwisting rate (in deg/s) was defined from the rotation velocity waveform as the first negative peak occurring during isovolumetric relaxation (Fig. 1). Speckle tracking was also used to measure the systolic peak of LV global longitudinal strain (GLS), and the peak of global early diastolic strain rate (Esr) obtained from each of the three apical views.

### DIVPG assessment by color Doppler M-mode

The non-invasive estimation of instantaneous DIVPGs by color Doppler M-Mode has been validated in animal and human studies [10, 11]. The investigators simplified the intracardiac pressure-velocity relationship by assuming that gravitational potential energy and energy losses due to viscous dissipation are both negligible. If the M-mode scanline approximates a flow streamline, it turns out that the Euler momentum equation yields the spatiotemporal pressure-velocity relationship along the M-line:

$$ \frac{\partial P}{\partial s}=-\rho \left[\frac{\partial v}{\partial t}+v\frac{\partial v}{\partial s}\right] $$

where P(s,t) and v(s,t) stands for pressure and Doppler velocity, respectively. The coordinate s represents the affine (spatial) coordinate along the streamline, and t is time. Density *ρ* is constant since blood is incompressible in the investigated conditions. This simplified equation shows that the pressure gradient (∂P/∂s) is the result of inertial (∂v/∂t) and convective (∂v/∂s) components [15]. Integration of this equation along the M-line, from the LV base to apex, yields an estimation of the DIVPG expressed by the unsteady Bernoulli equation:

$$ {P}_{base}-{P}_{apex}=\frac{1}{2}\rho \left({v}_{apex}^2-{v}_{base}^2\right)+\rho {\int}_{base}^{apex}\frac{\partial v}{\partial t} ds $$

**Fig. 2:**
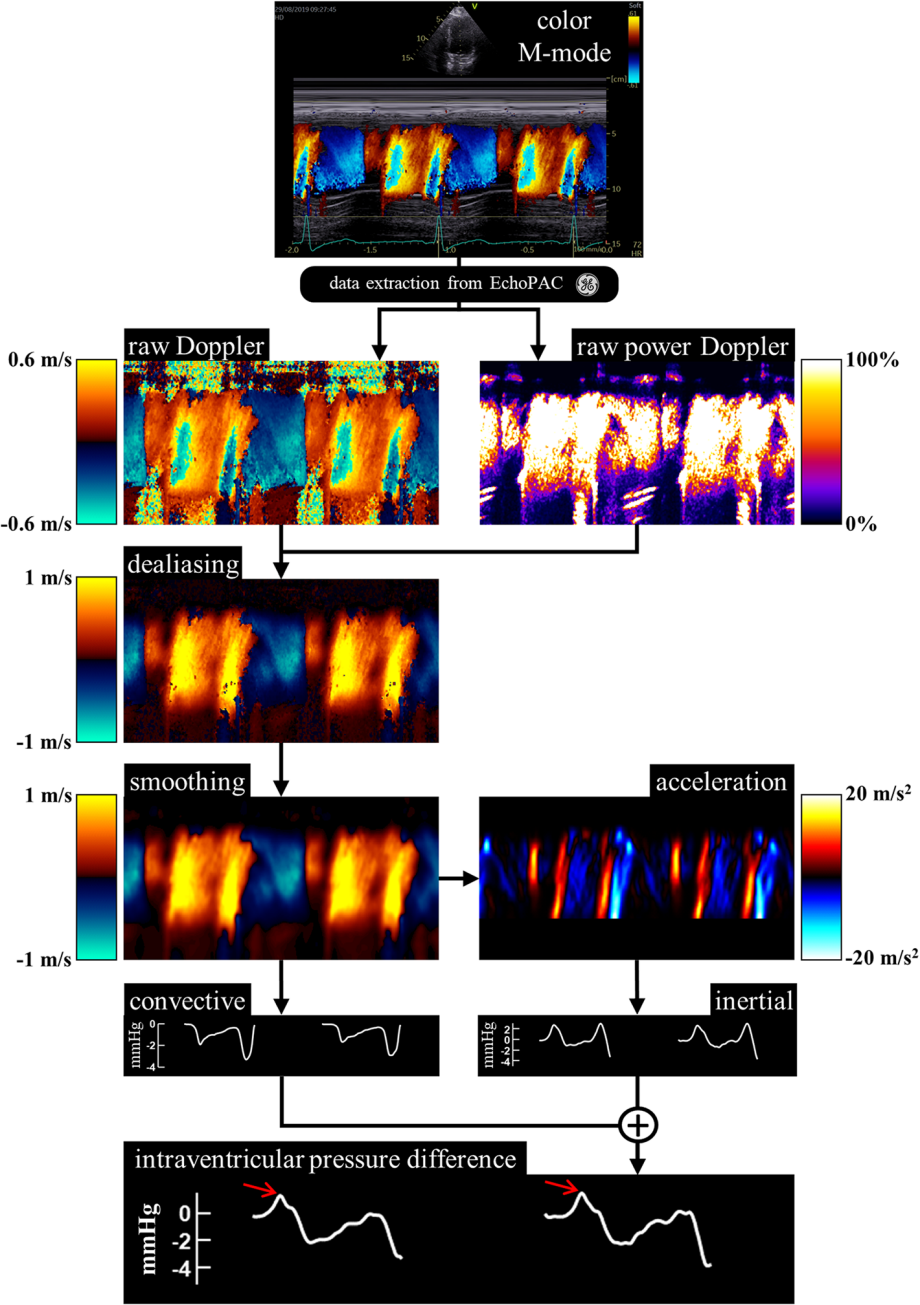
Semi-automatic assessment of peak DIVPG using color Doppler M-mode echocardiograms post-processed with custom software

Of note, this equation was successfully used and validated in aortic stenosis to estimate the instantaneous transvalvular pressure difference [16]. Based on the unsteady Bernoulli equation, all the color Doppler M-Mode tracings acquired along the base-to-apex axis using a standard apical four-chamber view could be used for our study. Three consecutive cardiac cycles were collected. The raw DICOM color Doppler M-Mode images were transferred to hierarchical h5 data files using EchoPAC. The images were then processed off-line using our homemade Matlab program to estimate the instantaneous pressure difference between the LV base and apex. The basal and apical levels were located manually by the operator. The numerical algorithm was then fully automatic and included unsupervised dealiasing and smoothing [17, 18] to ensure high inter-operator reproducibility. The peak pressure differences were determined during early filling for three successive heart cycles and averaged. The flowchart in Fig. 2 illustrates the sequence of pre- and post-processing used to assess peak DIVPG.

### Statistical analysis

Statistical analysis was performed with MedCalc Statistical Software (version 13.2.0, MedCalc Software, Belgium). The results were expressed as mean ± standard deviation if normally distributed, if not, as median ± interquartile range. Linear regression analyses were used to study the relationships between echocardiographic and clinical data. A multivariate linear regression analysis was used to determine the potential independent predictors of peak untwisting rate and peak DIVPG. The multivariate model was developed with stepwise inclusion and exclusion at a significant level of 0.1. The measurements during load changes were analyzed using a paired sample t-test. Test-retest reproducibility of DIVPG acquisitions was determined on two independent color Doppler M-mode measures made by the same operator for intra-observer variability, and by two independent operators on the same subject for inter-observer variability. We used the intraclass correlation coefficient with a 95% confidence interval and the absolute difference between repeated measurements expressed in percent of their mean. The level of significance was defined as *P* <  0.05.

## Results

### Population characteristics

Among 223 subjects meeting the inclusion and exclusion criteria, DIVPG and LV untwisting rate analyses were both feasible in 154/223 (69%) subjects included in the study. Exclusions were mainly due to poor speckle-tracking quality of the LV endocardium in short-axis at the basal or apical level preventing untwisting rate assessment in 60/223 (27%) subjects. DIVPG analysis was not feasible in only 9/223 (4%) subjects because of either an important misalignment of Doppler beam with trans-mitral inflow [19] or an incorrect delimitation of LV basal and apical levels. The population of the study consisted of 32 patients scanned for cardiovascular risk assessment, and 122 healthy volunteers screened for non-specific symptoms or pre-competitive physical exercise evaluation. The clinical characteristics of the population are summarized in Table 1*.* Seventy-four percent of the participants had no medical history. LV morphological and functional echocardiographic parameters measured in our entire population are reported in Table 2.

**Table 1:** Demographic and clinical parameters of the population (*n* = 154)

Parameters	Sample (%)	Median [25^e^-75^e^]	Range
Male	97 (63)		
Age (yrs)		23 [21–37.5]	18–77
Height (cm)		177 [170–183]	126–206
Weight (kg)		82 [70.2–93.3]	43.2–144
Body mass index (kg/m^2^)		26.6 [23.9–29.6]	17.5–41.1
Body surface area (m^2^)		2 [1.8–2.1]	1.2–2.7
Heart rate (bpm)		60.5 [54–69]	37–100
Systolic blood pressure (mmHg)		120 [111–128]	94–187
Diastolic blood pressure (mmHg)		72 [65–80]	52–105
Exercise training	88 (57.1)		
male	58 (65.9)		
age (yrs)		22 [20–23]	18–31
Arterial hypertension	20 (13)		
male	11 (55)		
age (yrs)		53.5 [47–61.5]	22–77
Diabetes	4 (2.6)		
Hyperlipidaemia	7 (4.5)		
Other medical history			
immune disease	5 (3.2)		
Non cardiac transplantation	2 (1.3)		
Chronic viral infection	2 (1.3)		

Values are expressed as median ± interquartile [25^e^-75^e^ percentile], and range

**Table 2:** Echocardiographic analysis

Variable	Mean ± SD	Median [25^e^-75^e^]	Range
Morphology			
LVIDd (mm)	50.4 ± 5.1		34–65
LVIDd/BSA (mm/m^2^)	25.6 ± 3.1		17.7–39.5
IVSd (mm)	8.6 ± 1.7		5–14
PWTd (mm)	8 ± 1.4		5–11
LV mass (g)		144 [115.3–168]	70.6–219
LV mass/BSA (g/m^2^)		70.1 [61.1–78.8]	43.5–108
LA volume (ml)	47.8 ± 14.6		36–59
LA volume/BSA (ml/m^2^)	24.3 ± 6.6		19.2–29
LV ejection fraction (%)		60 [55–65]	50–72
LV global longitudinal strain (%)	19.6 ± 2		15.3–25.3
Diastolic function			
Doppler			
Peak transmitral E-wave (cm/s)	72.2 ± 13.6		33–105
Peak transmitral A-wave (cm/s)		39 [32–48]	22–97
E/A ratio	1.87 ± 0.61		0.66–3.33
Peak early diastolic tissue velocity e’ (cm/s)	12.8 ± 2.9		5–18.5
E/e’ ratio		5.6 [4.8–6.6]	2.6–15
Speckle tracking			
Peak untwisting rate (deg/s)	68.5 ± 19.4		28.3–130
Peak early diastolic strain rate Esr (s^−1^)	1.45 ± 0.32		0.67–1.62
Color Doppler M-mode			
Peak DIVPG (mmHg)	3.2 ± 1		1.6–7.7
Peak Vp (cm/s)	77.6 ± 22.8		38–143
E/Vp ratio	0.99 ± 0.3		0.3–1.7

Values are expressed as mean ± SD if normally distributed or median and interquartile [25^e^-75^e^ percentile]. BSA: body surface area, *DIVPG* diastolic intraventricular pressure gradients, *IVSd* interventricular septal thickness in diastole, *LA* left atrial, *LV* left ventricle, *LVIDd* LV internal diameter in diastole, *PWTd* posterior wall thickness in diastole, *Vp* flow propagation velocity

### Test-retest reproducibility of DIVPG acquisitions

Test-retest reliability of DIVPG was determined in 20 subjects randomly selected from the echocardiographic database. For intra-observer reproducibility, the intraclass correlation coefficient was 0.97 [0.91–0.99], and the relative percentage difference was 4.28 ± 4.52%. For inter-observer reproducibility, the intraclass correlation coefficient was 0.97 [0.67–0.99], and the relative percentage difference was 8.96 ± 5.28%.

### Relationships between early diastolic functional parameters

**Fig. 3:**
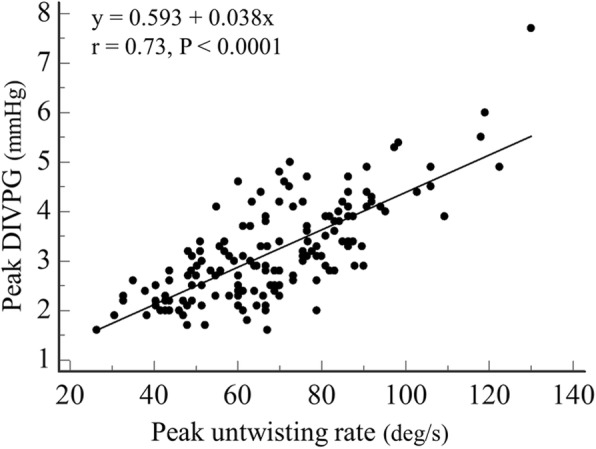
Relationship between peak DIVPG and peak untwisting rate in the entire population

**Fig. 4:**
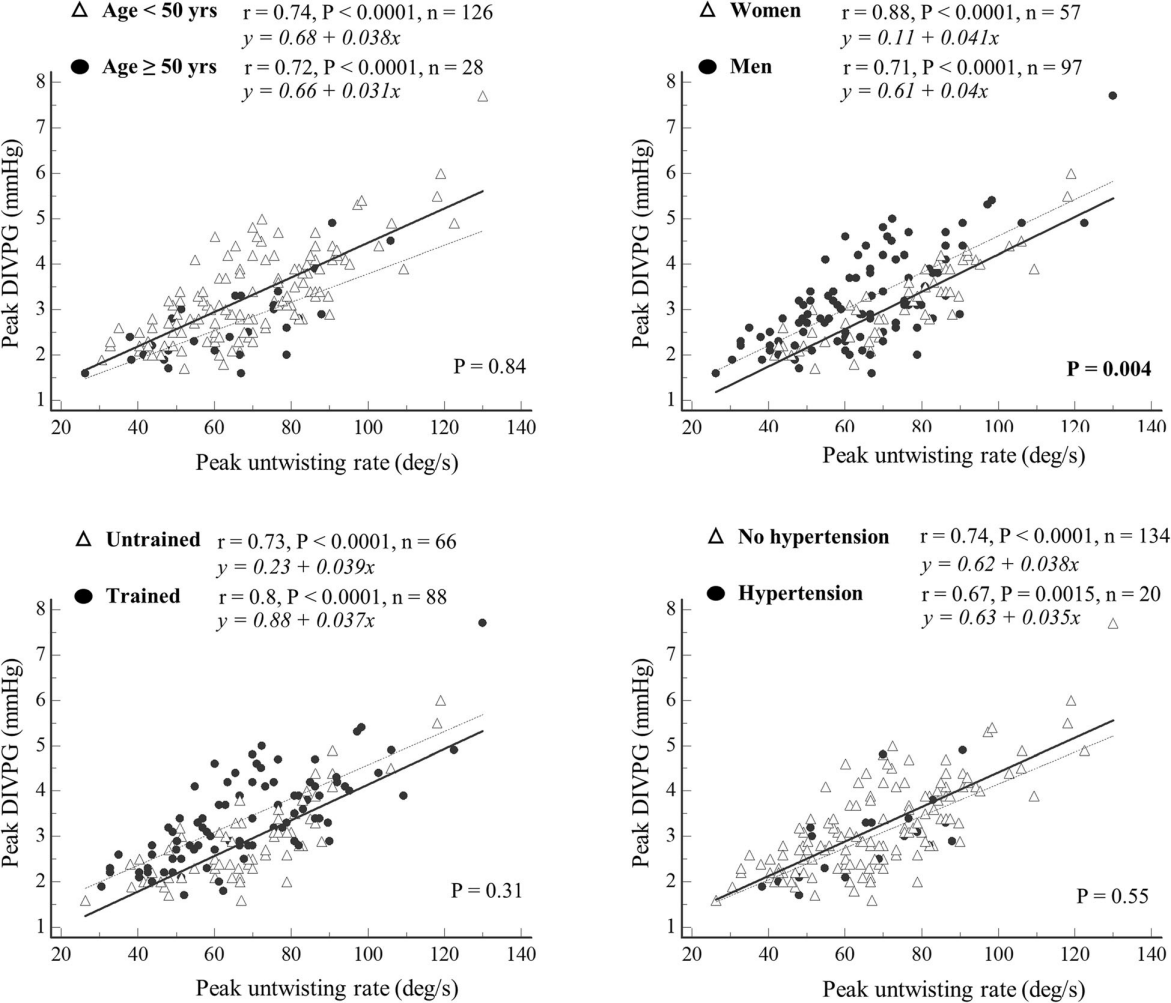
Relationship between peak DIVPG and peak untwisting rate depending on age, gender, training status, and personal history of hypertension The comparison of correlation coefficients was analyzed using Fisher’s Z-transformation.

**Fig. 5:**
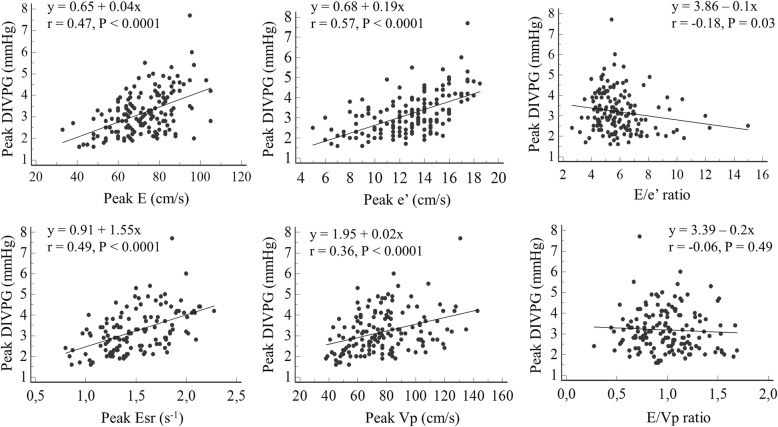
Correlations between peak DIVPG and the standard parameters of left ventricular early diastolic function and filling

**Fig. 6:**
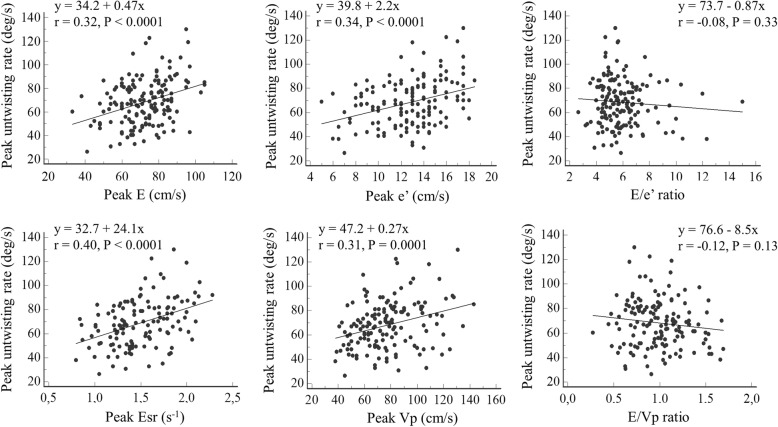
Correlations between peak untwisting rate and the standard parameters of left ventricular early diastolic function and filling

There was a positive and significant correlation between peak DIVPG and peak untwisting rate (r = 0.73; *P* <  0.001) in our population (Fig. 3). This correlation was maintained regardless of age (≤ 50 years old or > 50 years old), gender, exercise training status, and personal history of hypertension (Fig. 4). Correlation between DIVPG and untwisting rate was slightly stronger in women compared to men (Fig. 4). Peaks E, e’, Esr and Vp showed poor to moderate correlations with both peak DIVPG and peak untwisting rate (Figs. 5 and 6). Only peak DIVPG was inversely and significantly correlated with E/e’ ratio. LA volume was not correlated with peak untwisting rate (*P* = 0.09). Multivariate analysis revealed that peak DIVPG was the only variable that was independently associated with peak untwisting rate (β-coefficient = 14.1 ± 2, R^2^-adjusted = 0.53, *P* <  0.0001).

### Clinical factors that influence untwisting rate and DIVPG

Univariate analysis showed that peak untwisting rate significantly decreased with aging, that peak untwisting rate differed significantly between males and females (74.6 ± 19.2 deg/s for females vs. 64.9 ± 18.7 deg/s for males, *P* = 0.002), and that peak untwisting rate was inversely associated with body mass index (Table 3). Multivariate analysis revealed that only age and sex were independently associated with peak untwisting rate (Table 3). As shown in Table 4, peak DIVPG was associated with age and chronic exercise training on univariate analysis, but age was the only variable that was independently associated with peak DIVPG on multivariate analysis. Peak DIVPG decreased with aging. There was no difference in peak DIVPG between men and women (3.17 ± 0.9 mmHg vs. 3.21 ± 1.1 mmHg, *P* = 0.81). The median age was similar between sexes (27 yrs. [22–47.5] for women vs. 23 yrs. [21–33] for men, *P* = 0.12).

**Table 3:** Results of univariate and stepwise multivariate analysis for untwisting rate

Variable	Univariate analysis				Multivariate analysis			
	Coefficient (Slope)	Std Error	r_partial_	*P*	Coefficient (Slope)	Std Error	r_partial_	*P*
Age	−0.21	0.1	− 0.16	0.05*	− 0.25	0.1	− 0.2	0.015
Sex	−9.7	3.1	− 0.24	0.002*	−11.2	3.1	−0.28	0.0005
Body mass index	−0.69	0.32	−0.17	0.03*	−0.43	0.32	−0.11	0.19
Heart rate	0.064	0.13	0.04	0.62				
Systolic blood pressure	0.11	0.11	0.08	0.34				
Diastolic blood pressure	0.042	0.15	0.02	0.78				
Exercise training	0.14	3.2	0.004	0.96				

* Variables with a P-value < 0.1 in univariate analysis were included in the multivariate analysis

**Table 4:** Results of univariate and stepwise multivariate analysis for diastolic intraventricular pressure gradients

Variable	Univariate analysis				Multivariate analysis			
	Coefficient (Slope)	Std Error	r_partial_	*P*	Coefficient (Slope)	Std Error	r_partial_	*P*
Age	−0.02	0.01	−0.36	< 0.0001*	−0.24	0.005	− 0.36	< 0.0001
Sex	0.04	0.17	0.02	0.81				
Body mass index	0.014	0.017	0.07	0.4				
Heart rate	−0.006	0.007	−0.07	0.37				
Systolic blood pressure	0.002	0.006	0.03	0.74				
Diastolic blood pressure	−0.01	0.008	−0.11	0.17				
Exercise training	0.51	0.16	0.25	0.002*	−0.03	0.22	− 0.01	0.89

* Variables with a P-value < 0.1 in univariate analysis were included in the multivariate analysis

### Passive leg raising test

**Fig. 7:**
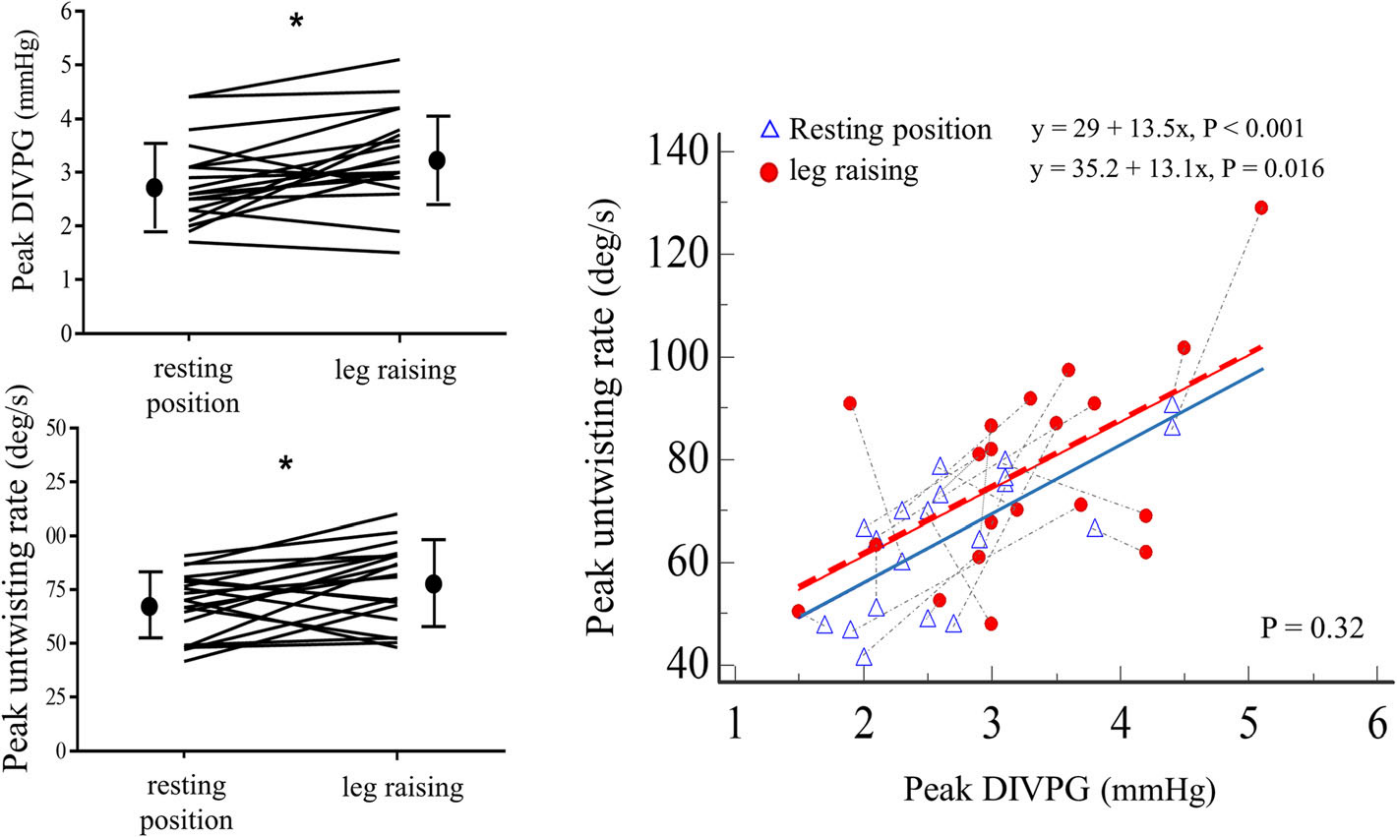
Relationship between DIVPG and untwisting rate during a passive leg raising maneuver. Mean peaks DIVPG and untwisting rate increased significantly after a passive leg raising in 20 healthy volunteers (left, *P < 0.001), without altering the correlation between the magnitudes of peak untwisting rate and peak DIVPG (right). Each pair of resting and leg raising states was connected by a line

During passive leg raising maneuver performed in 20 healthy male volunteers, we observed simultaneously significant variations of mean DIVPG (2.71 ± 0.77 mmHg vs. 3.25 ± 0.88 mmHg, *P* < 0.001) and untwisting rate (65.5 ± 14.2 deg/s vs. 77.1 ± 20.5 deg/s, *P* = 0.011), with inter-individual variability (Fig. 7). Correlations between untwisting rate and DIVPG were not significantly different before and after leg raising (*P* = 0.32). The heart rate was slightly but significantly increased after leg raising (65 [58–71] bpm vs. 68.5 [59–75] bpm, *P* = 0.03).

## Discussion

The present work has confirmed on a large adult population with preserved cardiac function a close relationship between the amplitudes of peak DIVPG and peak untwisting rate. This relationship remained strong regardless of age, gender, exercise training, chronic hypertension, and acute changes in LV load did not alter it. Although DIVPG is independently associated with untwisting rate and explains much of its variability, these two parameters of LV early diastolic function are complementary but not interchangeable as the former is related to blood flow and the latter to the myocardium.

DIVPG quantification by color Doppler M-mode provides a robust approach to assess early diastolic intraventricular inflow through the measure of the instantaneous velocity distribution. In contrast to the alias-based slope method for measuring Vp responsible for lower feasibility and reproducibility [9], which was weakly correlated with peak DIVPGs and untwisting rate, DIVPG assessment allows a more comprehensive evaluation of LV suction. Echocardiographic acquisition and calculation of DIVPGs are uncomplicated, it requires a single standard apical four-chamber view, and the use of automatic post-processing ensures low test-retest variability and high technical feasibility, which was confirmed in the present study on a large adult cohort. Our software using velocity data before scan conversion could potentially be implemented on a work station and used in any clinical echo lab.

First human studies that have demonstrated the relationship between LV untwisting (assessed by tissue Doppler imaging) and DIVPGs were conducted on small groups of healthy volunteers (*n* = 20) and patients with hypertrophic cardiomyopathy (*n* = 7) [12, 13]. LV untwisting precedes DIVPG genesis which promotes LV suction work. In the temporal sequence of diastolic filling, these two mechanisms resulting from restoring forces precede peak early filling and LV expansion [1, 13]. In line with these observations, our study confirmed in a large adult cohort without heart disease a similar good correlation between the magnitudes of peak DIVPG and peak untwisting rate assessed by speckle tracking. Our multivariate analysis showed that peak DIVPG was independently associated with peak untwisting rate. Due to this strong interplay between DIVPG and untwisting rate, DIVPG could provide a reliable (96% of feasibility versus only 73% for untwisting rate) and less time-consuming (one apical acquisition automatically post-processed) evaluation of LV early diastolic recoil. Peak untwisting rate was shown to be associated not only with LV relaxation rate but also with LV restoring forces [5]. Studies on intraventricular pressure gradients have also confirmed a close relationship with both LV relaxation rate and contractility [3, 4, 20]. Significant but weaker correlations for untwisting rate and DIVPGs were observed with early-diastolic filling velocity (assessed by E) and myocardial lengthening velocity (assessed by e’ and Esr). Although LV filling is related to many factors, including left atrial function and venous return, the association between LV restoring forces and early filling could be expected through LV suction [1, 12, 13]. LV early-diastolic lengthening velocity was shown to be determined by LV suction, in addition to LV lengthening load and stiffness [21]. Nonetheless, e’ and Esr are region-dependent parameters, which limits the assessment of the LV base-apex velocity gradient.

In this study, age similarly affected the untwisting rate and DIVPG. We found that age was an independent predictor for both peak untwisting rate and peak DIVPG. Therefore, the relationship between untwisting rate and DIVPG remained similar in younger and older subgroups. Impairment in LV diastolic function is part of the aging process of the heart, which affects myocardial intrinsic relaxation proprieties and disturbs cellular calcium homeostasis [22]. Previous works have demonstrated that untwisting rate is delayed with aging, which in turn affects LV active early filling and intraventricular pressure gradients [23, 24]. Our results confirmed a decrease in peak untwisting rate and peak DIVPG with aging. The relationship between untwisting rate and DIVPG was slightly but significantly better in women compared to men. In our population, male sex seemed to independently and negatively affect peak untwisting rate, the latter being greater in women. Previous observations have reported that females exhibited greater LV rotation and faster untwisting velocity following acute preload reduction and inotropy stimulation [25], suggesting an adaptive mechanism to generate adequate intraventricular pressure gradients and filling and to balance gender-related differences in LV geometric and myocardial intrinsic functional proprieties [26].

Previous observations in healthy volunteers have suggested that the load dependency of LV untwisting rate may be a physiological compensatory mechanism to minimize the effects of load variations on LV filling and stroke volume [27, 28]. Experimental findings have confirmed that acute changes in LV early diastolic load have direct effects on untwisting rate, in addition to LV relaxation rate and restoring forces [5]. Popovic et al. [29] showed that variations in DIVPGs assessed by color Doppler M-mode observed during acute load manipulations were related to changes in pulmonary capillary wedge pressure and were modulated by LV relaxation rate. We used passive leg raising as a simple and safety maneuver to evaluate the effects of a transient cardiac load variation on the relationship between untwisting rate and DIVPG. Our results are consistent with a load dependency of these two parameters assessed by echocardiography, without any significant change observed in their relationship.

### Limitations

The first limitation is related to the techniques used for non-invasive assessment of intraventricular pressure gradients and untwisting rate. Since color Doppler is a one-dimensional imaging technique (only velocity vector projections are returned), flow needs to be coaxially interrogated by the ultrasound M-mode scan-line so as not to underestimate blood velocity. Thus, we cannot preclude some misalignment in the course of this study. However, based on our past experience with this approach, the DIVPG calculation is only marginally or not affected by a slight misalignment of the Doppler scanline with respect to the flow streamline [19]. Moreover, although special care was taken to ensure that the peak untwisting rate was measured as previously described, we did not assess the exact time of opening of the mitral valve [7]. Secondly, passive leg raising is commonly used to increase cardiac preload. It is also known to influence central blood pressure via baroreceptor activation in healthy subjects [30]. We did not measure the early diastolic load increment nor the potential effects of sympathetic activation that might have led to some of the inter-individual variability in the untwisting rate and DIVPG measurements. We cannot also predict whether the effects of passive leg raising would be similar in females. Untwisting rate response to preload reduction was shown to be greater in females than males but with no significant difference following preload augmentation by saline infusion [25]. Finally, our trained population was much younger than sedentary subjects. With the understanding of the determinant effect of age on both DIVPGs and untwisting rate that we have shown, this selection bias might explain that chronic exercise training was not associated with parameters of LV active relaxation in our study.

## Conclusions

DIVPG measured by color Doppler M-mode was well correlated and independently associated with LV untwisting rate. Their interdependence indicates a strong physiological coupling between the intraventricular fluid forces and the myocardium relaxation. This echocardiographic approach to DIVPG estimation, highly feasible and reproducible, could improve the assessment of LV diastolic function. Our team is currently working on the development of turnkey clinical software for computing DIVPG, to further study its diagnostic value in patients with heart diseases.

## Data Availability

The datasets used and/or analyzed during the current study are available from the corresponding author on reasonable request.

## Abbreviations

A: Peak velocity of late filling wave
DIVPG: Diastolic intraventricular pressure gradient.
E: Peak velocity of early filling wave.
e’: Mitral annulus early peak velocity.
Esr: Peak of global early diastolic strain rate.
GLS: Global longitudinal strain.
LA: Left atrial.
LV: Left ventricle.
LVEF: Left ventricular ejection fraction.
Vp: Color M-mode Doppler flow propagation velocity.
